# A Grating Interferometric Acoustic Sensor Based on a Flexible Polymer Diaphragm

**DOI:** 10.3390/s23249912

**Published:** 2023-12-18

**Authors:** Linsen Xiong, Zhi-mei Qi

**Affiliations:** 1 State Key Laboratory of Transducer Technology, Aerospace Information Research Institute, Chinese Academy of Sciences, Beijing 100190, China; xionglinsen17@mails.ucas.ac.cn; 2 School of Electronic, Electrical and Communication Engineering, University of Chinese Academy of Sciences, Beijing 100049, China

**Keywords:** optical acoustic sensor, grating interferometry, flexible diaphragm

## Abstract

This study presents a grating interferometric acoustic sensor based on a flexible polymer diaphragm. A flexible-diaphragm acoustic sensor based on grating interferometry (GI) is proposed through design, fabrication and experimental demonstration. A gold-coated polyethylene terephthalate diaphragm was used for the sensor prototype. The vibration of the diaphragm induces a change in GI cavity length, which is converted into an electrical signal by the photodetector. The experimental results show that the sensor prototype has a flat frequency response in the voice frequency band and the minimum detectable sound pressure can reach 164.8 µPa/√Hz. The sensor prototype has potential applications in speech acquisition and the measurement of water content in oil. This study provides a reference for the design of optical interferometric acoustic sensor with high performance.

## 1. Introduction

Optical interferometric acoustic sensors have been widely studied recently and have advanced applications in areas such as sound source localization [1,2,3], speaker recognition [4] and medical devices [5,6]. The most dominant acoustic sensors are based on the principle of capacitive detection. The performance of capacitive acoustic sensors is limited by the scaling law. In contrast, optical interferometric acoustic pressure detection methods are immune to the scaling laws as electrical methods [7]. Optical interferometric acoustic sensors are mainly based on Sagnac interferometer (SI) [8], Fabry-Perot interferometer (FPI) [9,10,11], laser self-mixing interferometer (LSMI) [12], and grating interferometer (GI) [13,14]. The common implementation of the SI and the FPI requires optical fibers, which makes it difficult to integrate [15]. The LSMI has a high degree of integration. However, due to the complexity of its demodulation algorithm, the LSMI is not suitable for detecting high-frequency acoustic signals and is limited to detecting infrasound [16]. Compared to other optical interferometric acoustic sensors, GI-based acoustic sensors could be designed with high integration, wide bandwidth, and high acoustic fidelity [17,18].

According to previous studies, the diaphragm materials used to construct GI acoustic sensors are monocrystalline silicon [19,20], polycrystalline silicon [21,22], and silicon nitride [23]. In order to have wide bandwidth for the acoustic sensor, the diaphragm needs to be properly tensile pre-stressed. Methods to adjust the tensile pre-stress of silicon-based diaphragms include annealing, doping, and stress compensation [24,25]. The way to apply tensile pre-stress to flexible polymer diaphragms is relatively simple. Due to the flexibility of the polymer diaphragm, the diaphragm tensile pre-stress can be applied by mechanical stretching [26]. Therefore, a GI acoustic sensor based on a flexible polymer diaphragm is proposed and experimentally demonstrated in this study. The flexible diaphragm is made of polyethylene terephthalate (PET). The PET diaphragm is coated with a metal film underneath, which, together with the fabricated metal grating, forms the GI. The vibration of the diaphragm introduces a change in the GI cavity length, which is detected by a photodetector (PD) and output as a voltage signal. The performance of the fabricated GI acoustic sensor is also theoretically simulated and experimentally tested. The subsequent section describes the principle, design, fabrication and experimental results of the proposed optical interferometric acoustic sensor.

## 2. Principle and Design

**Figure 1:**
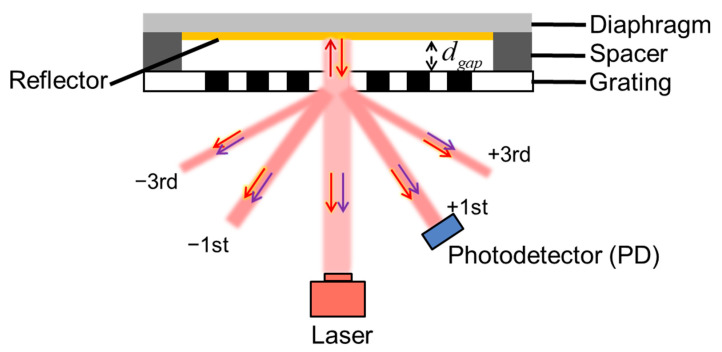
Schematic diagram of the grating interferometer (GI)-based acoustic sensor.

The general structure of the GI-based acoustic sensor is schematically shown in Figure 1. The sensor is composed of a diaphragm, spacer, grating with 1/2 duty ratio, laser and PD. The diaphragm and grating form the GI with a cavity length of $d_{gap}$. The initial $d_{gap}$ is determined by the thickness of the spacer. When the sensor operates, the laser is emitted by a laser diode (LD) or vertical cavity surface emitting laser (VCSEL). The laser first reaches the grating surface and is then split into two parts. One part is reflected by the grating surface. The other part enters the GI cavity and is reflected back to the grating by the diaphragm. Both parts diffract on the grating surface. The part that enters the GI cavity travels a longer optical path than the part that diffracts directly from the grating. As a result, optical interference occurs below the grating surface, and a series of diffraction spots (0th, ±1st, ±3rd, etc.) are produced. According to the scalar diffraction theory, the normalized interferometric optical intensities of the 0th and ±1st diffraction spots are as follows:

(1) $$\frac{I_{0}}{I_{in}}=\frac{1}{2}\left[1+\cos\left(\frac{4\pi d_{gap}}{\lambda }\right)\right]$$

(2) $$\frac{I_{\pm 1}}{I_{in}}=\frac{2}{\pi^{2}}\left[1-\cos\left(\frac{4\pi d_{gap}}{\lambda }\right)\right]$$

where $I_{0}$ and $I_{\pm 1}$ are the interferometric optical intensities of the 0th and ±1st diffraction spots, respectively, $I_{in}$ is the intensity of the laser emitted from the LD or VCSEL, and λ is the wavelength of the laser. Since it is necessary to reflect the laser, a reflector is uniformly plated or coated on the lower surface of the diaphragm to increase the reflectivity. The reflector is usually a thin metal film.

When acoustic pressure is applied to the GI acoustic sensor, the vibration of the diaphragm causes a change of $d_{gap}$. According to the vibration theory [27,28], the displacement amplitude $d_{r}$ and the first resonant frequency $f_{1}$ of the circular diaphragm are as follows:

(3) $$d_{r}=\frac{p_{in}\rho }{\omega^{2}P_{d}{}^{2}h}\left[\frac{J_{0}(kr)}{J_{0}(kr_{d})}-1\right]$$

(4) $$f_{1}=\frac{2.405}{2\pi r_{d}}\sqrt{\frac{P_{d}}{\rho }}$$

where r is the radial variable, $p_{in}$ is the amplitude of sound pressure, $J_{0}$ is the zero-order column Bessel function, ω is the angular frequency of vibration, $P_{d}$ is the magnitude of the radial tensile pre-stress, $r_{d}$, h, ρ are the radius, thickness, density, of the diaphragm, respectively, the tension is $T_{d}=P_{d}h$, and $k=\sqrt{\omega^{2}P_{d}/\rho }$ is the wave number. When $kr_{d}<<1$, the displacement at the center of the diaphragm $d_{0}$ is:

(5) $$d_{0}=\frac{p_{in}r_{d}{}^{2}}{4P_{d}h}$$

which is theoretically the change in $d_{gap}$. According to Equations (1) and (2), the change in $d_{gap}$ causes a variation of the interferometric optical intensity. The optical intensity is detected by the PD and then converted to a current signal. Therefore, the acoustic pressure can be measured by the electrical signal output from the PD. Considering the spatial location and diffraction efficiency, the ±1st diffraction spot was chosen as the detection signal in the actual sensor design.

First resonance frequency and mechanical sensitivity are important indicators of an acoustic sensor. The first resonant frequency is usually designed to be higher than the operating band range to give the sensor a wide operating bandwidth. And, mechanical sensitivity is usually used to define the magnitude of the mechanical response of the diaphragm to sound pressure. According to Equation (3), the mechanical sensitivity $S_{m}$ is:

(6) $$S_{m}=\frac{d_{r}}{p_{in}}$$

Theoretically, the GI acoustic sensor detects the displacement at the center of the diaphragm. Therefore, according to Equations (5) and (6), mechanical sensitivity is:

(7) $$S_{m0}=\frac{r_{d}{}^{2}}{4P_{d}h}$$

**Figure 2:**
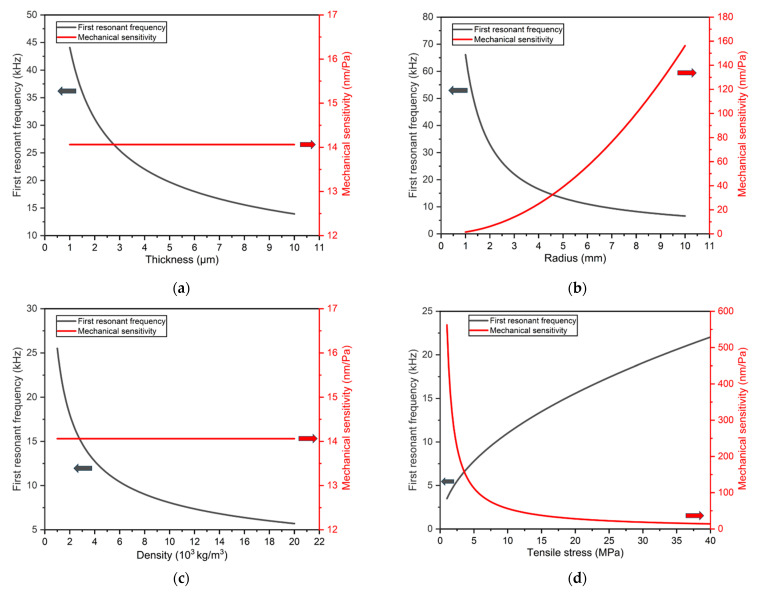
Theoretical analysis of the first resonant frequency and mechanical sensitivity for different (**a**) thickness, (**b**) radius, (**c**) density, (**d**) tensile pre-stress of diaphragm.

In order to design a diaphragm with a higher first resonant frequency and higher mechanical sensitivity, the influence of diaphragm thickness, radius, density, and tensile stress on performance is analyzed based on Equations (4) and (7). The analysis results are shown in Figure 2. Figure 2a indicates that when the applied tension to the diaphragm is fixed, a thinner diaphragm thickness leads to a higher first resonant frequency. Moreover, the change in diaphragm thickness has no effect on the mechanical sensitivity. However, since the linear deformation range of the diaphragm is 30% of its thickness [29], a thinner diaphragm results in a smaller sound pressure measurement range. Therefore, the design of the diaphragm thickness still requires a trade-off consideration. Figure 2b shows that as the radius increases, the first resonant frequency and mechanical sensitivity of the diaphragm exhibit opposite trends. In practical applications, diaphragms are typically manufactured as standard acoustic sensors with package sizes of 1/2 inch, 1/4 inch, and so on. Therefore, this study chooses to design the diaphragm radius as 3mm for the production of common 1/2-inch acoustic sensors. Figure 2c indicates that a lower diaphragm material density leads to a higher first resonant frequency. Therefore, a higher first resonant frequency can be designed by selecting an appropriate diaphragm material. Figure 2d shows that a larger tensile stress results in a higher first resonant frequency but also leads to a lower mechanical sensitivity. Therefore, the magnitude of the tension applied to the diaphragm is determined by the design objectives of the acoustic sensor.

Considering the above design factors, PET has been chosen as the material for the diaphragm. This type of flexible polymer materials is widely used in the production of flexible devices [30]. The materials used in previous studies for the diaphragms of GI acoustic sensors are nickel, monocrystalline silicon, polycrystalline silicon, and silicon nitride. Compared to these materials, PET has a lower density, as shown in Table 1. Therefore, with the same diaphragm size and tensile pre-stress, the diaphragm made of PET has a higher first resonance frequency according to Equation (4) and Figure 2c.

**Figure 3:**
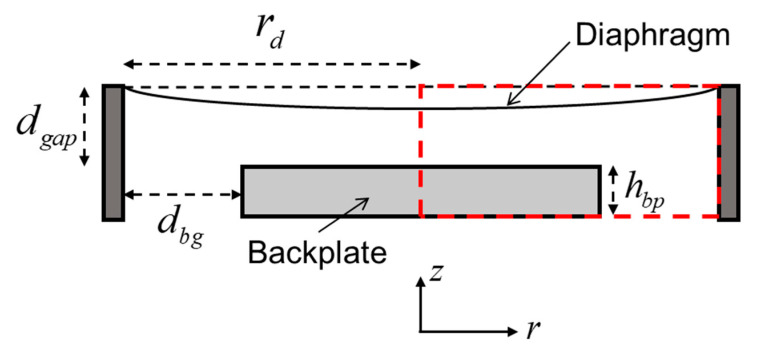
Sketch of the acoustic sensor including variables and coordinate system. The red dashed box indicates the modeled region.

In order to conduct further analysis of the mechanical performance of the designed sensor, the basic characteristics of the diaphragm structure were simulated using the finite element method. The structural diagram used in the simulation is shown in Figure 3. The modeling area is shown in the red dashed box. In order to make effective use of computational resources in simulation, a two-dimensional axisymmetric model is created, and the red dashed box in Figure 3 shows the modeling area. The parameters of the simulated structure are summarized in Table 2. As shown in Table 2, based on the theoretical analysis presented in Figure 2, the simulation parameter for the diaphragm thickness is selected as 4 μm in order to achieve a good dynamic range for the diaphragm. Then, in order to observe the response of the diaphragm in the low-frequency band, the parameter of tensile pre-stress should be set to a larger value, which is set to 40 MPa in this case. Additionally, considering the use of a 0.5 mm thick glass plate as the backplate in subsequent experiments, the backplate thickness is set to 0.5mm in the simulation.

**Figure 4:**
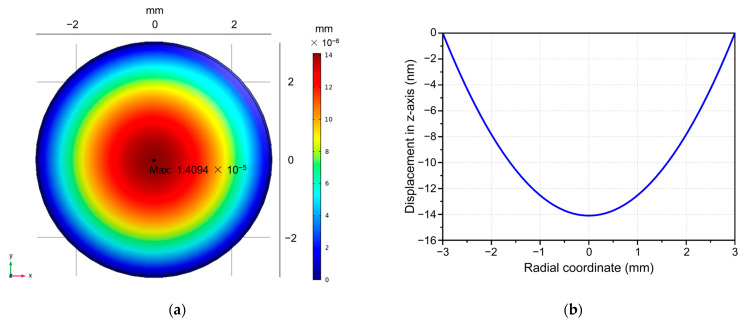
Simulated deformation of the diaphragm at a sound pressure of 1 Pa at 1 kHz. (**a**) Displacement distribution in the plane of the diaphragm. (**b**) Displacement distribution on the radial direction of the diaphragm (z-component).

Figure 4 shows the simulated variation in diaphragm deflection with applied sound pressure (1 Pa) at 1 kHz. It is worth noting that the displacement in the center region of the diaphragm is larger than the displacement in the edge region. The center of the diaphragm has a maximum deflection of 14.094 nm. According to Equation (5), the theoretical value of the diaphragm center displacement is 14.062 nm, which is in good agreement with the simulated value. Additionally, the linear deformation range of the diaphragm is 30% of its thickness, which is 1.2 μm. Therefore, the maximum linear measurement range of the PET diaphragm for sound pressure is approximately 85.1 Pa.

**Figure 5:**
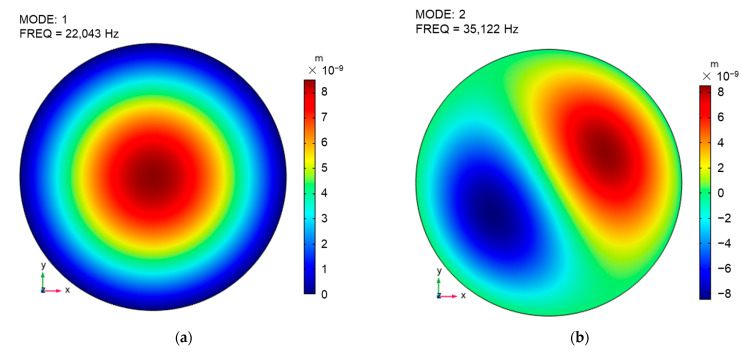

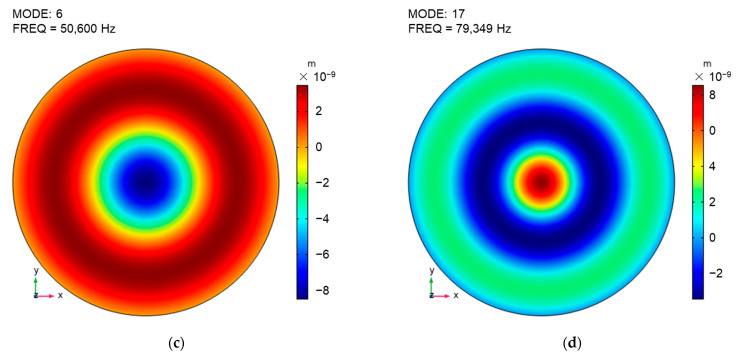
Simulated mode shapes and corresponding modal frequencies of the diaphragm: (**a**) mode 1, (**b**) mode 2, (**c**) mode 6, (**d**) mode 17.

Figure 5 shows the simulation results of modal analysis performed on the diaphragm. Figure 5a–d respectively presents the mode shapes and corresponding frequencies of mode 1, mode 2, mode 6, and mode 17. Among them, the frequency corresponding to mode 1 is usually the first resonance frequency. Therefore, the first resonance frequency obtained from the simulation is 22.043 kHz, which is consistent with the value (22 kHz) calculated by Equation (4). It is worth noting that, compared to the subsequent frequency response analysis simulations, the frequency of mode 1 obtained from modal analysis can more accurately calculate the first resonance frequency with fewer computational resources.

**Figure 6:**
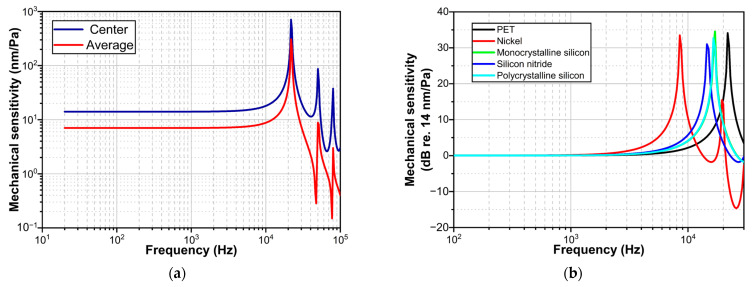
(**a**) Simulated frequency response curve of mechanical sensitivity. (**b**) Simulated frequency response curves of mechanical sensitivity of diaphragms (center point) of different materials.

Figure 6 shows the results of the frequency response analysis simulation. Considering that the amplitude of the input sound pressure is 1 Pa, the corresponding displacement can be converted into mechanical sensitivity. A comparison of the average value of the mechanical sensitivity of the diaphragm with the center value is shown in Figure 6a. Figure 6a shows that the center value of the diaphragm displacement is larger than the average value in the frequency range of 20 Hz to 100 kHz. The curves in the range of ±3 dB fluctuation with the mechanical sensitivity at 1 kHz as a reference were defined as flat frequency band. In the flat frequency band, the center value (14.094 nm/Pa) of mechanical sensitivity is about twice the average value (7.031 nm/Pa). Moreover, the capacitive-based displacement detection principle usually detects the average displacement of the whole diaphragm, while the GI-based displacement detection principle detects the center displacement. Therefore, the mechanical sensitivity of the GI acoustic sensor is twice that of the capacitive acoustic sensor when the same diaphragm is used. In addition, Figure 6 shows three frequency resonance peaks, with corresponding frequencies of approximately 22 kHz, 50 kHz, and 80 kHz, respectively. Combined with the analysis of the modal shapes results, it can be deduced that the three frequency resonance peaks correspond to mode 1, mode 6, and mode 17 presented in Figure 5. Through the analysis results of Figure 5 and Figure 6, it can be found that the acoustic sensor is not suitable for detecting wide-band sound pressure signals above the frequency of mode 1 due to the changes in different modal shapes. Therefore, in order to achieve wide-band signal detection, the sensor should operate within the flat frequency band.

Frequency response curves were also obtained for diaphragms of different materials displaced at the center point, as shown in Figure 6b. Figure 6b shows that the mechanical sensitivity at the center point of the diaphragm for all five materials in the flat frequency band is about 14 nm/Pa. Thus, the mechanical sensitivity is independent of the material density, which is consistent with Equation (4). In addition, the first resonant frequency of the PET diaphragm is the highest among the diaphragms of these five different materials. This is due to the fact that the density of PET is the lowest among the five materials.

**Figure 7:**
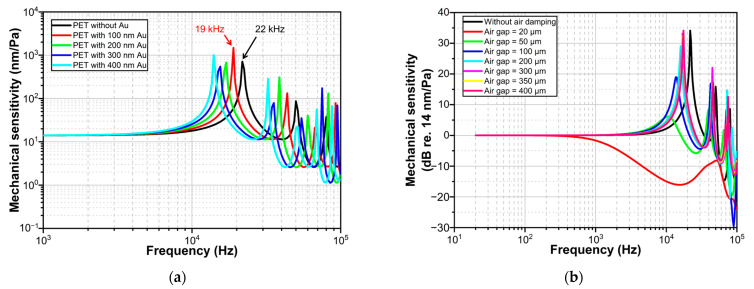
Simulated frequency response of diaphragm (center point) mechanical sensitivity for different thickness of: (**a**) gold reflector, (**b**) air gap.

Since thin PET diaphragms are usually transparent, it is necessary to coat a certain thickness of metal reflector on the diaphragm to increase its optical reflectivity. Considering the effect of the metal reflector on the mechanical response of the diaphragm, PET diaphragms coated with different thicknesses of metal reflector were simulated using the equivalent single layer theory [31]. In this simulation, the metal reflector used is gold, and its density is 19,300 kg/m^3^. The simulation results are shown in Figure 7a. Figure 7a illustrates that a thicker gold coating results in a lower first resonance frequency, but does not affect the mechanical sensitivity of the flat frequency band. A 100 nm thick Au layer causes the first resonance frequency of the PET to decrease from 22 kHz to 19 kHz. The composite density of 4 um thick PET coated with 100 nm thick Au is approximately 1778 kg/m^3^, which is greater than the density of PET. Therefore, according to Equation (4), the first resonance frequency of this composite structure is 18.9 kHz, which is close to the simulated value. As the thickness of the gold plating increases, the composite density further increases, resulting in a decrease in the first resonance frequency. Therefore, a thinner metal reflector should be used while ensuring that the diaphragm has a certain optical reflectivity.

The diaphragm vibration discussed above does not involve the influence of the surrounding air. In fact, the vibration of the diaphragm will drive the air vibration and is also damped by the air. The effect of air damping cannot be easily ignored. In order to evaluate the effect of air damping, the frequency response of the mechanical sensitivity at the center of the diaphragm is simulated for different thicknesses of the air gap, as shown in Figure 7b. Figure 7b shows that the presence of an air gap has an effect on the first resonant frequency of the diaphragm. A small air gap (e.g., 20 μm) results in a reduction in the flat frequency band. A suitable air gap (e.g., 50 μm) can widen the flat band. A large air gap (e.g., 400 μm) reduces the effect of air damping on the resonance frequency. Therefore, in order to obtain a good frequency response, it is necessary to design an air gap.

**Figure 8:**
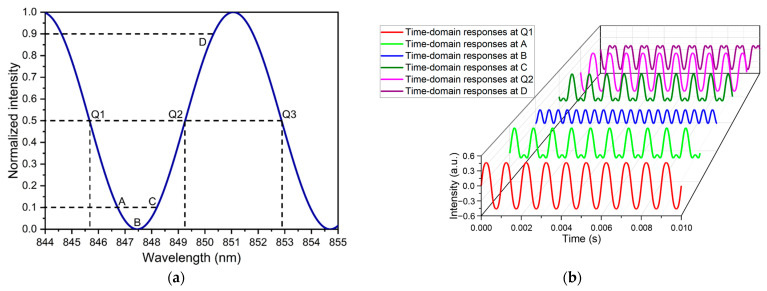
(**a**) Optical interference curve of the GI-based acoustic sensor. (**b**) The responses of GI-based acoustic sensor work at different operating points.

In addition to the analysis of the diaphragm, a further analysis of the grating interferometer was also conducted. Considering the position and optical utilization, the optical intensity of the first diffraction spot was chosen as the detection signal. The optical interference curve of the first intensity analyzed based on Equation (2) is shown in Figure 8a. The cavity gap selected for analysis is 50 μm. The optical curve is sine-like in the case of the monochromatic laser beam. It can be found that the sensor’s operating point depends on the wavelength when the cavity gap is fixed. The responses of the GI-based acoustic sensor operating at different operating points of the interference curve are analyzed with the signal frequency of 1 kHz. The analysis results are shown in Figure 8b. It can be observed that the responses at different operating points are different in one cycle. The responses are distorted when the acoustic sensor works at points A, B, C, and D. It should be noted that the response of the sensor operating at point B is completely distorted. Therefore, the sensor should work in the linear range centered at quadrature points (Q1, Q2 & Q3) for a high-fidelity output signal.

The cavity gap usually has a machining error during the fabrication of the sensor [14,19,32]. Thus, the initial operating point is uncertain. The operating point can be adjusted by tuning the wavelength of the LD, which can make the GI-based acoustic sensor work to achieve maximum high fidelity. According to Figure 8a, the half cycle of the optical interference curve is 3.6 nm (from 847.5 nm to 851.1 nm). Thus, the LD selected for building sensors with a cavity length of 50 μm should have a wavelength tuning range over 3.6 nm.

## 3. Fabrication and Packaging

**Figure 9:**
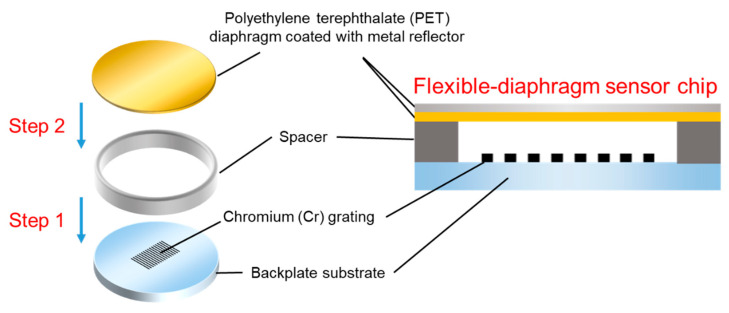
Schematic diagram of the fabrication process of the proposed flexible-diaphragm acoustic sensor chip.

**Figure 10:**
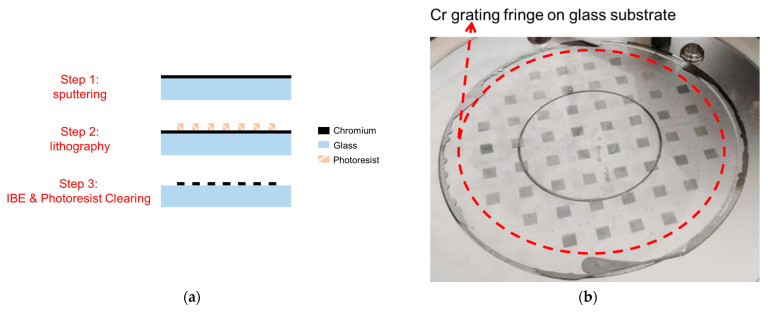

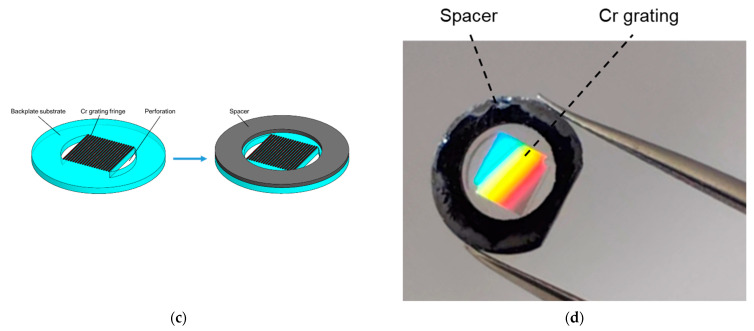
(**a**) Fabrication process of the chromium grating on the glass substrate. (**b**) Photograph of the grating on the glass substrate after ion beam etching. (**c**) Schematic diagram of the combination of grating and spacer. (**d**) Photograph of the “grating-spacer” structure.

Figure 9 shows a schematic of the fabrication process of the proposed flexible-diaphragm acoustic sensor chip. The fabrication process consists of two main steps. Firstly, the fabrication process of the chromium (Cr) grating on the backplate substrate has been detailed in Figure 10a. A 50 nm Cr film for grating fabrication was sputtered onto a high-transparency glass substrate [33]. Then, a photoresist mask with grating patterns was formed on the Cr film using lithography technology. Afterwards, the Cr film not covered by the photoresist was removed through ion beam etching (IBE). The wafer after IBE is shown in Figure 10b, where the grating pattern has been formed on the glass substrate. And, the grating period is 2.4 μm. Next, the photoresist was cleaned off to retain the Cr film with the grating pattern. Subsequently, perforations were formed on the glass substrate using laser cutting, and a silicon wafer was cut to form spacers. The glass substrate and silicon spacers were assembled together using anodic bonding, as schematically shown in Figure 10c. Finally, the wafer after anodic bonding was subjected to laser cutting to form individual “grating-spacer” structures, as illustrated in Figure 10d.

**Figure 11:**
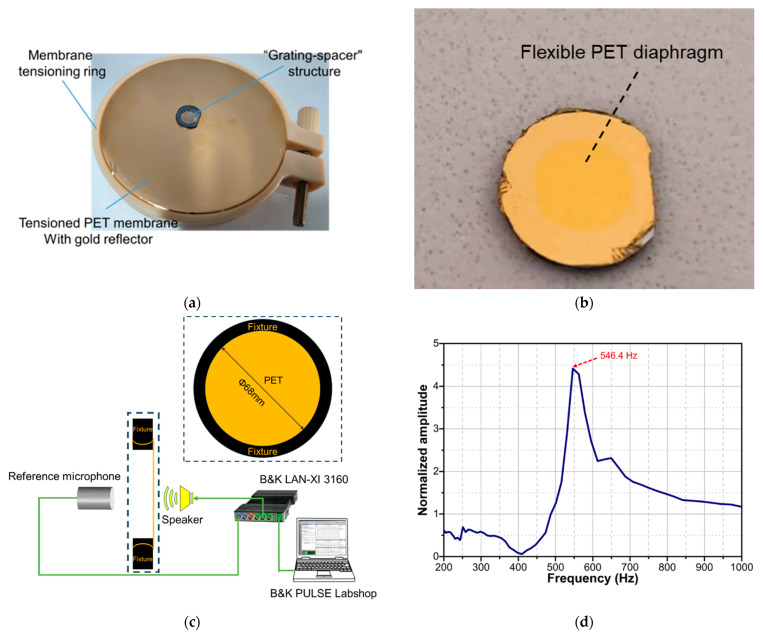
(**a**) Photograph of assembly of PET membrane with “grating-spacer” structure. (**b**) Photograph of proposed flexible-diaphragm acoustic sensor chip. (**c**) Schematic diagram of experimental setup for testing resonant frequency of tensioned large-size membrane. (**d**) Resonant frequency of the tensioned large-size membrane.

According to Figure 9, the second step involves assembling the PET diaphragm with the “grating-spacer” structure. First, a 100 nm thick gold reflector was deposited on a large-size PET membrane with a thickness of 4 μm using magnetron sputtering technology. Then, a large-size PET membrane was pre-tensioned using membrane tensioning ring [26], which is a method of applying pre-stress through mechanical stretching. The pre-tensioning force was applied to the PET membrane with a diameter of 68 mm. Next, the “grating-spacer” structure was tightly adhered to the large-size PET membrane using a UV adhesive, as shown in Figure 11a. Finally, the small-size PET diaphragm with a “grating-spacer” structure was cut out using a blade. The small-size PET diaphragm with an effective radius of 3 mm is shown in Figure 11b. In this manufacturing process, the tension of the pre-tensioned PET membrane with a diameter of 68 mm can be measured using the resonant method [34]. The experimental setup for tension measurement is shown in Figure 11c, and its principle is to derive the tensile pre-stress through the measured resonant frequency of the large-size membrane using Equation (4). The test result of the membrane with a diameter of 68 mm are shown in Figure 11d. Figure 11d shows that the resonant frequency of the membrane is 546.4 Hz, and thus indicates that the tension applied to the pre-tensioned PET membrane is 3.16 MPa. Therefore, according to Equations (4) and (7), the small-size PET diaphragm with an effective radius of 3 mm, cut from the large-size membrane, has a theoretical first resonant frequency of 6.2 kHz and a mechanical sensitivity of 178 nm/Pa. Since the maximum linear deformation of the 4 μm thick PET diaphragm is 1200 nm, the maximum measurable sound pressure of this PET diaphragm is about 6.7 Pa.

**Figure 12:**
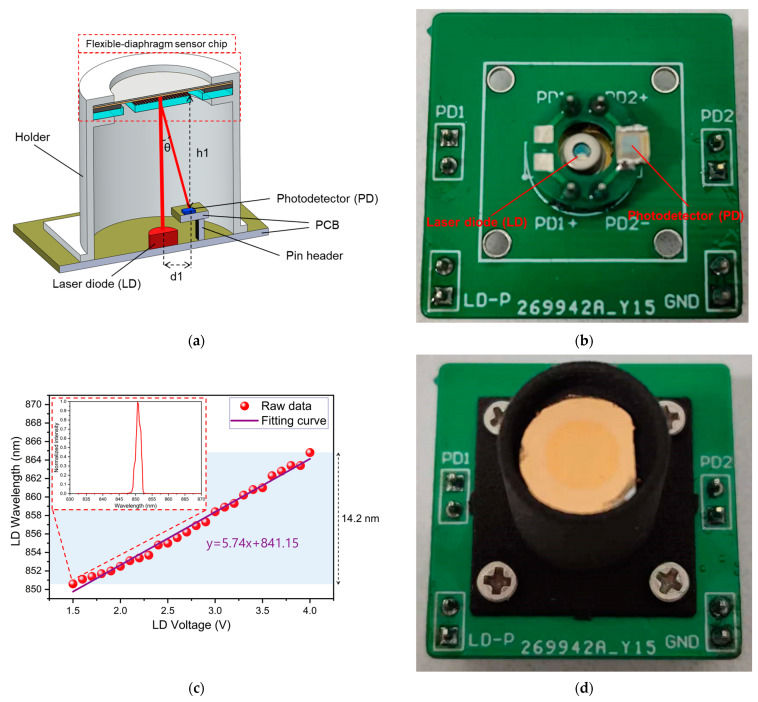
(**a**) Schematic diagram of the packaged acoustic sensor. (**b**) Photograph of the laser diode (LD) and photodetector (PD) utilized in the work. (**c**) Measured wavelength range of the LD. (**d**) Photograph of the packaged acoustic sensor.

For experimental testing, the sensor chip was packaged. A schematic of the packaged acoustic sensor is shown in Figure 12a. The sensor chip was placed on top of the holder, while the diaphragm was exposed to perceive sound pressure, as shown by the red dashed box in Figure 12a. The central area of the diaphragm was aligned with the optical window of the LD. A PD was placed on one side of the LD to receive the diffracted spot. Based on the theory of diffraction, the distance (d1) between the center of the LD and the center of the PD, as well as the height (h1) from the surface of the PD to the surface of the grating was determined by the angle (θ) of the first diffraction order of the grating. Both the holder and optical components were mounted on a printed circuit board (PCB), which interfaced with a peripheral circuit. This peripheral circuit consisted of a driving electrical module connected to the LD and a signal processing module connected to the PD. The output voltage signal of the signal processing module was then used to detect the acoustic signal. In this work, the infrared LD (Model D6-6-850-50, Egismos Inc., Chinese Taipei, Taiwan region) was utilized as the laser source, as depicted in Figure 12b. The wavelength tuning range of the LD, measured through a spectrometer (Model AQ6370B, Yokogawa Inc., Tokyo, Japan), is shown in Figure 12c. Figure 12c shows that the wavelength varies by 14.2 nm as the LD voltage changes from 1.5 V to 4 V. The inset illustrates the laser spectrum measured by the spectrometer at a driving voltage of 1.5 V, indicating a wavelength of 850.6 nm. The large tuning range of wavelengths allows the sensor to operate at the quadrature working point. The photograph of the sensor chip and its packaging is presented in Figure 12d.

## 4. Experiment and Discussion

**Figure 13:**
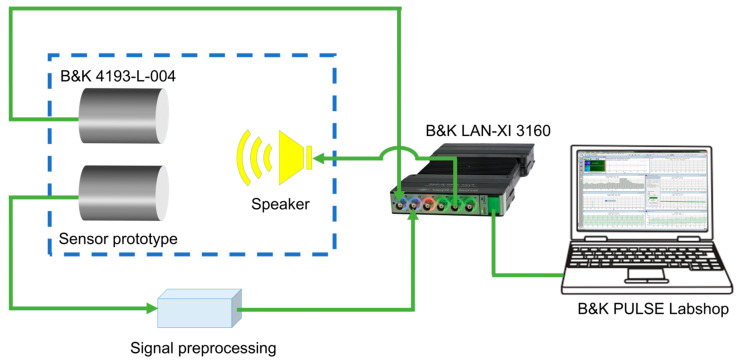
The experimental setup for acoustic characterization.

To characterize the packaged acoustic sensor prototype, an acoustic test system was built, as shown in Figure 13. The test system consists of the sensor to be tested, a signal processing module (powered by a lithium battery), a reference acoustic sensor, a loudspeaker, a multifunctional module and signal analysis software. The proposed sensor and a reference acoustic sensor (B&K 4193-L-004) are placed side by side in front of a loudspeaker, as shown by the blue dashed box in Figure 13. The signal generator of the multifunctional module (B&K LAN-XI 3160) drives the loudspeaker to produce a single-frequency acoustic signal with a certain sound pressure. A grating interferometric acoustic sensor excited by the sound pressure outputs a photocurrent signal. The photocurrent signal is converted to a voltage signal by a signal processing module and then acquired by a multifunctional module. The output signal of the reference acoustic sensor is acquired simultaneously. The acquired signals are transferred to a PC with the electroacoustic equipment test system software (B&K PULSE Labshop Version 21.0.0.671) and analyzed by the software.

**Figure 14:**
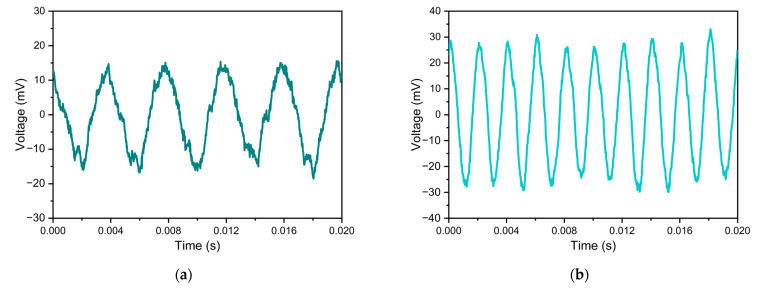

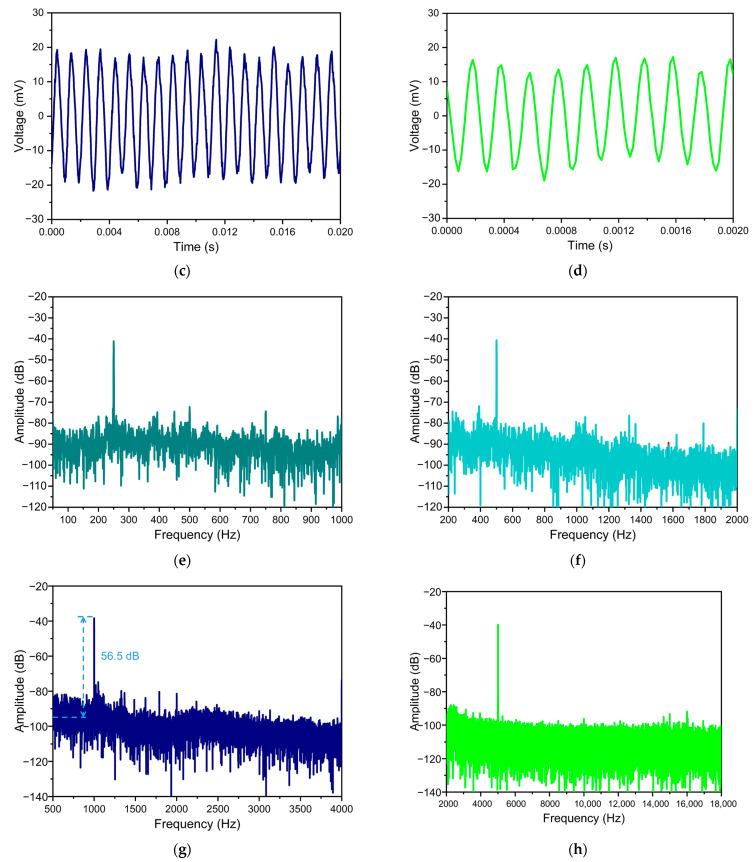
Responses of the proposed sensor to acoustic signals with different frequencies and corresponding frequency domain results obtained by Fourier transform processing. (**a**,**e**) 250 Hz; (**b**,**f**) 500 Hz; (**c**,**g**) 1000 Hz; (**d**,**h**) 5000 Hz.

The single-frequency response of the sensor was tested based on the experimental setup as shown in Figure 14. Sinusoidal acoustic waves with frequencies of 250 Hz, 500 Hz, 1.0 kHz, and 5.0 kHz were generated, respectively. The time domain signals of the sensor are shown in Figure 14a–d, and the corresponding frequency domain spectra are shown in Figure 14e–h. It can be seen that the main peaks of the FFT spectra corresponding to the signal waveforms at each frequency are obvious, and the main peak frequencies are consistent with the corresponding test acoustic frequencies. In addition, the minimum detectable sound pressure (MDP) is a key performance of the acoustic sensor. MDP can be calculated from the FFT spectrum. In Figure 14g, the applied 1 kHz acoustic waveform is 77.9 mPa. For a frequency resolution of 0.5 Hz, the background noise is about −94.8 dB, and the measured SNR is 56.5 dB. Therefore, the calculated MDP is 164.8 µPa/√Hz.

**Figure 15:**
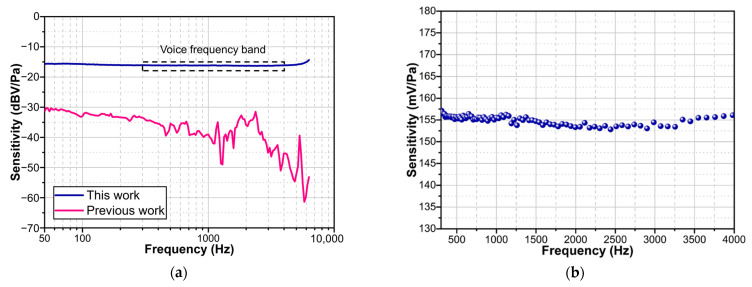
(**a**) Frequency response curve from 50 Hz to 6.4 kHz. The previous work is Ref. [13]. (**b**) Frequency response test points over voice frequency band.

In addition to the MDP, a flat frequency response is a key performance of the acoustic sensor. The frequency response of the proposed acoustic sensor was tested from 50 Hz to 6.4 kHz. The test results are shown in Figure 15a. The response of the sensor proposed in this work is flat in the range of 50 Hz to 6.4 kHz with a fluctuation range of no more than 3 dB. This frequency response performance is a significant improvement over the previous GI-based acoustic sensor [13,35]. And the prototype in [13] is based on a monocrystalline silicon diaphragm. Due to the brittle of monocrystalline silicon diaphragm, it is impossible to apply tensile pre-stress through mechanical stretching [36]. Meanwhile, applying pre-stress on monocrystalline silicon via micro-electro-mechanical system (MEMS) technology remains a relatively complex technique [24]. Compared to monocrystalline silicon, PET possesses flexibility and stretchability, allowing for a simpler method to apply tensile pre-stress, and thus, offers a better frequency response than uncontrolled pre-stressed monocrystalline silicon diaphragm [13]. The response test points of the acoustic sensor in the voice frequency band (300 Hz to 4.0 kHz) are shown in Figure 15b. As shown in Figure 15b, the sensitivity at 1 kHz is 155.6 mV/Pa. The sensitivity does not fluctuate by more than 3.2% in the voice frequency band, which indicates that the sensor has potential applications in situations related to speech acquisition [37,38].

In addition, the permeability of water and oil to PET is very low; thus, the acoustic sensor with PET as the diaphragm has potential applications in the measurement of water content in oil [39]. And various polymers can be further investigated for achieving high performances of flexible-diaphragm acoustic sensor. There may be other polymer materials made diaphragms that enable the sensor to have a wider frequency band or lower MDP [40,41].

## 5. Conclusions

In conclusion, a flexible PET diaphragm acoustic sensor has been proposed based on the GI. Firstly, the measurement principle of GI and the theory of membrane vibration are analyzed and explained. The size, density, and pre-stress of the diaphragm are theoretically analyzed to illustrate the impact of these design parameters on sensor performance. Meanwhile, the PET diaphragm is modeled and analyzed using finite element simulation. The simulation results provide design guidance for the metal coating and air gap of the diaphragm. Furthermore, by analyzing the response of GI at different operating points, a wavelength-tuning method is employed to control the operating point. Then, the flexible diaphragm sensor chip is fabricated by MEMS process and machining. The sensor chip was packaged in a prototype for experimental testing. The experimental results show that the packaged sensor prototype can reach a minimum detectable sound pressure of 164.8 µPa/√Hz and a sensitivity of 155.6 mV/Pa at 1 kHz. Moreover, the sensor prototype has a flat frequency response with a fluctuation of no more than 3.2% in the voice frequency band, which has potential application in speech acquisition. And, the low water and oil permeability of the PET diaphragm suggest a potential application of the sensor in measuring water content in oil. Based on this study, the future goal is to achieve the overall flexibility of the sensor, improve system integration, and expand its application to wearable devices.

## Figures and Tables

**Table 1:** Properties of diaphragm materials.

Material	PET	Nickel	Monocrystalline Silicon	Polycrystalline Silicon	Silicon Nitride
Density (kg/m^3^)	1340	8800	2300	2330	3000

**Table 2:** Parameters of the simulated model.

Description	Parameter	Value or Range
(Fixed) Radius of diaphragm	$r_{d}$	3 mm
(Fixed) Thickness of diaphragm	h	4 μm
(Fixed) Tensile pre-stress of diaphragm	$P_{d}$	40 MPa
(Variable) Air gap	$d_{gap}$	20~400 μm
(Fixed) Thickness of backplate	$h_{bp}$	0.5 mm
(Fixed) Amplitude of sound pressure	$p_{in}$	1 Pa

## Data Availability

Data are contained within the article.
